# Diabetes Risk by Length of Residence among Somali Women in Oslo Area

**DOI:** 10.1155/2016/5423405

**Published:** 2016-05-25

**Authors:** Abdi A. Gele, Kjell Sverre Pettersen, Bernadette Kumar, Liv Elin Torheim

**Affiliations:** ^1^Oslo and Akershus University College of Applied Sciences, P.O. Box 4, Street Olavs Plass, 0130 Oslo, Norway; ^2^Norwegian Center for Minority Health Research, P.O. Box 4956, Nydalen, 0424 Oslo, Norway

## Abstract

Type 2 diabetes represents a major health problem worldwide, with immigrants strongly contributing to the increase in diabetes in many countries. Norway is not immune to the process, and immigrants in the country are experiencing an increase in the prevalence of diabetes after arrival. However, the dynamics of these transitions in relation to the duration of residence in the new environment in Norway are not clearly understood. From this background, a cross-sectional quantitative study using a respondent-driven sampling method was conducted among 302 Somali women living in Oslo area. The results show that 41% of the study participants will be at risk for developing diabetes in the coming 10 years, which coincides with 85% of the study participants being abdominally obese. Significant associations were found between years of stay in Norway and the risk for diabetes with those who lived in Norway >10 years, having twofold higher odds of being at risk for developing diabetes compared to those who lived in Norway ≤5 years (OR: 2.16, CI: 1.08–4.32). Understanding the mechanisms through which exposure to the Norwegian environment leads to higher obesity and diabetes risk may aid in prevention efforts for the rapidly growing African immigrant population.

## 1. Background

Diabetes is increasing globally at an alarming rate, becoming one of the most costly and burdensome chronic diseases of our time. According to the International Diabetes Federation (IDF), 8.3% of the world's adults (382 million people) have diabetes, and the number is set to rise beyond 592 million within the next two decades [1]. Type 2 diabetes (T2D) accounts for 90–95% of all diabetes. Among immigrant communities, an epidemic of overweight and obesity, a consequence of loss of traditional lifestyles and the adaptation of unhealthy behavior, has been noted [2]. Not only do migrant groups develop T2D at a younger age, but they also have higher morbidity and mortality from T2D and related complications [1, 3]. The available data indicates that African immigrants in particular experience a high burden of diabetes [4–6]. Norway is not immune to the process, and despite the progress made in identifying diabetes risks and the health behaviors associated with it in different ethnic groups in Norway [7], there is scarcity of data among African immigrant women in Norway.

Researchers do not fully understand why some people develop T2D and others do not, though it is clear that certain factors increase the risk of developing the condition. Family history of diabetes has been shown to be a risk factor for type 2 diabetes, and individuals with a family history of diabetes mellitus are at a higher risk of developing the condition, thus reflecting the common behaviors and consequences of genetic susceptibility [8]. It is also worth noting that high blood pressure and diabetes are interlinked, as high blood pressure has the potential to increase the progression of prediabetes into type 2 diabetes [9]. Being from certain racial groups, such as Black Africans and Asians, also increases the susceptibility of T2D. The presence of impaired glucose tolerance (IGT), impaired fasting glucose, or both is called prediabetes, and it affects millions of overweight people [10], thereby raising their risk for developing diabetes. Nonetheless, an estimated 90% of T2DM is suggested to be attributable to lifestyle factors such as excess weight, sedentary behavior, and unhealthy diet [11]. Multiple studies have shown that an intensive lifestyle modification, including regular physical activity, healthy dietary choices, and modest weight loss are effective interventions among adults at high risk for developing type 2 diabetes [12, 13].

The early prevention of T2D or delaying its onset is the only opportunity available to curtail the growing diabetes epidemic; hence, people who are exposed to risk factors are often prioritized in diabetes prevention programs. However, the prevention of diabetes demands good access to health care educational programs and healthy food and opportunities to be physically active. Still, countries hosting immigrants are facing challenges in diabetes prevention among highly diverse immigrant communities [7]. This challenge partially stems from the fact that many immigrant communities have different concepts of health and health care than mainstream societies in the host country [14]. Therefore, to meet immigrant's preventive health needs, hosting countries need a greater understanding of the health status, health behaviors, and risk factors prevalent in different groups within the population. For example, a slim body is associated with poverty and ill health in many African cultures, while physical activity is perceived as something that is integrated into everyday lives; thus the promotion of physical activity by joining a fitness class or going for a long walk or jogging is not considered as useful or necessary [15]. In some communities, activities involving sweating and deep breathing are associated with negativity, whereas sitting and resting are associated with affluence. The language proficiency is another factor that affects immigrants' exposure to preventive services, with immigrants who do not speak the national language being less likely to benefit from community-based health promotion programs. Immigrants from sub-Saharan Africa were reported to not pay much attention to T2D, considering it something beyond an individual's control [14]. Therefore, due to misconceptions indicated by popular health beliefs, many Africans fail to take appropriate measures for the prevention and control of diabetes and its risk factors [16].

In 2015, immigrants comprised 15.6% of Norway's population, a percentage that is expected to increase rapidly [17]. The health and health services needs for this large and growing share of the population are not necessarily the same as those of mainstream Norwegian society. Research has repeatedly found a “healthy immigrant effect”—immigrants' health is generally better than that of the mainstream community—although it tends to decline as their years in the host country increase [18]. However, the relationship between immigration and health is complex, especially because the origins of immigrants to Norway are increasingly diverse, and they have different vulnerabilities to ill health.

In Norway, diabetes constitute a major public health problem, with 6 to 7,000 Norwegians acquiring the disease annually, while a similar number may remain undetected and thus unaccounted for [19]. However, the diabetes burden in Norway is more clustered toward immigrant communities [20]. For example, 90% of women from Pakistan who live in Norway were found to be at risk for diabetes [21]. Gele and Mbalilaki, who estimated the prevalence of diabetes risk factors among Somali immigrant women, found that the obesity pattern increased by the duration of stay in Norway, as evidenced by a higher body mass index (BMI) rate beginning after five years [22]. Although Somalia has the lowest prevalence of diabetes (4.3%) in the Arab region [23], an increased prevalence of diabetes was reported from Somali diaspora in the US [24]. Somali women are of particular concern, given their unique vulnerability in developing T2D. An increase in sedentary behavior, coupled with a drastic change in diet among Somali women, may lead to being overweight and acquiring diabetes [14]. Largely shaped by a lifelong deprivation of quality health systems in their home country, the exposure to war, and the experience of refugee life, Somalis were reported to focus on issues of immediate survival, with many of them having no reference for the notion of prevention and the long-term management of chronic diseases [25]. In the same vein, consulting with a general practitioner (GP) for chronic diseases screening and illness prevention is unfamiliar to most Somalis, who are accustomed to only seeking health care when ill [26]. Consequently, most chronic diseases are likely to go undetected until complications arise, with the reason for this being that no disease is perceived as a problem unless it is accompanied by symptoms that drive one to seek health care and warrant treatment, such as fever, diarrhea, pain, and cough.

The Norwegian diabetes strategy recognizes the significant disparities in knowledge on healthy eating and physical activity among communities, and reducing this disparity is seen as an important goal to help prevent diabetes [27]. Consequently, structural mechanisms involving activity-promoting residential areas, motivation of an active lifestyle and healthy eating through robust guidance, and population-based information and attitude shaping work were prioritized in the national diabetes strategy [27]. Although these recommendations are targeting the mainstream populations, it is apparent that most of them are also valid for immigrants, at least if certain modifications are made. Therefore, we explored the utilization of diabetes preventive measures among Somali immigrant women [28]. The study found that a nexus of social, structural, cultural, and economic barriers creates a pressure that makes it difficult for Somali women to utilize diabetes preventive health services [28]. The present study aims to examine the risk for diabetes by length of residence among Somali immigrant women in Oslo area.

### 1.1. Recruitment of Participants

Prior quantitative studies on African immigrants have used a nonprobability sampling design [22]. Because the sample frame of this immigrant community is rarely available or accessible, conventional methods of selecting a random sample for the cross-sectional surveys could not be applied. Moreover, the existing nonprobability sampling methods for immigrants, that is, snowball sampling, introduce well-documented sampling biases, whereby no statistical inference from the sample to the larger target population can be made with any accuracy [29]. Nevertheless, as postulated by the literature of the “small world,” this approach could potentially reach all members in the target population in only six waves [30], so total coverage is very likely with a resultant statistically invalid sample of broader coverage. As a result, we used respondent-driven sampling (RDS), which overcomes this dilemma through combining the wide coverage of network-based methods and the statistical validity of standard probability sampling methods [31]. RDS is a chain referral method, with the major difference between snowball sampling and RDS being that seeds recruit their peers, rather than identifying them to an investigator using a uniquely coded coupon [32]. This type of peer-to-peer recruitment removes any selection bias that may be created by the survey staff, while the use of an equal number of coupons for the recruitment minimizes biases associated with the overrepresentation of those participants within large networks [32]. In RDS, participants recruit their peers, as in network-based samples, and researchers keep track of who recruited whom and the number of social contacts of each respondent. A mathematical model of the recruitment process then weights the sample to compensate for nonrandom recruitment patterns [33].

### 1.2. Data Collection

**Figure 1:**
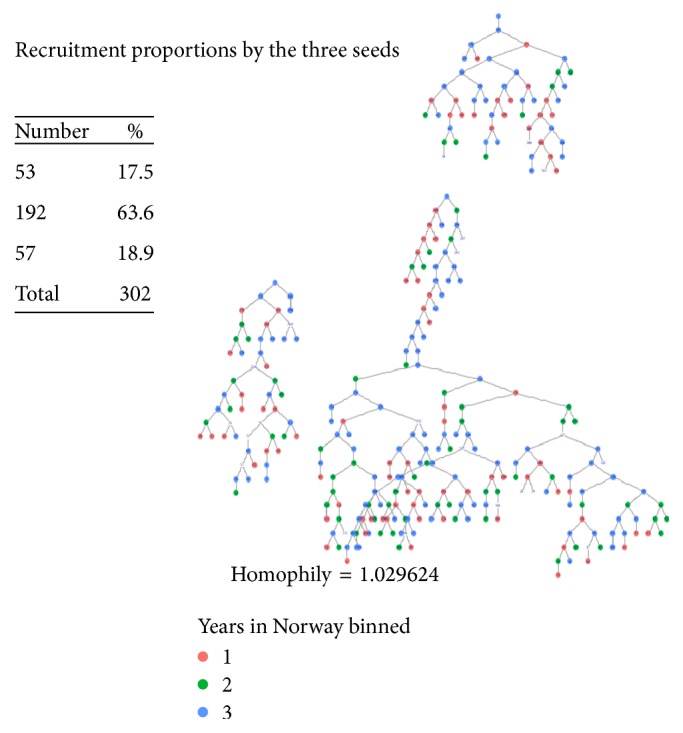
Recruitment chain by duration of stay in Norway.

Eligibility criteria for the study included being a Somali female, permanently and legally residing in Oslo and Akershus regions of Norway, age ≥25, and being willing to provide informed consent. Initially, a formative study involving five Somali women was conducted with the aim of understanding the social network structure of Somali women, the average peer that each recruiter can recruit, and the recruitment incentives necessary to motivate Somali women to participate in the study. A respondent-driven sampling design suggests that seeds should be persons who are socially connected and motivated to recruit others [31]. For this reason, three eligible seeds, comprising socially well-connected women, were purposely selected based on a diversity of location, years of stay in Norway, and age. After providing informed consent, the seeds underwent an interview, were educated on how to refer other eligible Somali women, and were given two uniquely coded coupons to help refer their peers in their social network. The reason for only using two coupons was to elongate the recruitment waves so that the diversion of subsequent waves from the initial seeds was increased. Each seed proceeded to recruit two persons into their network, which became the first wave, with the first-wave participants further recruiting their peers, which then became the second wave. Recruitment chain by years of residence in Norway is indicated in Figure 1. Each participant was compensated with 100 Norwegian kroner (NOK) for participating in the study and received an additional 100 NOK for each recruited peer who met the eligibility criteria and participated in the study. The chain referral process continued until we obtained the desired sample size of 300, which was calculated using the formula necessary to determine the sample size required for estimating the sample proportions. The first author and research assistant familiar with the Somali community in Oslo/Akershus primed the community in early 2014 about the upcoming study while waiting for ethical clearances. Data collection office containing two rooms was hired at Gronland neighborhood of Oslo, an area predominantly inhabited by the target population. Data collection including anthropometric measurements was conducted at the data collection center. A Somali-female research assistant, two volunteers (a male and a female), and the first author have collected the data. The study was ethically approved by the Norwegian Data Registry (NSD).

## 2. Outcome of Interest

The main outcome variable was age-adjusted diabetes risk among adult women of Somali descent. We used the Finnish Diabetes Risk Score (FINDRISC), which estimates the risk of developing diabetes within the next 10 years [10], which can be accessed here (http://www.idf.org/webdata/docs/FINDRISC_English.pdf).

The following eight FINDRISC items were used to estimate the level of diabetes risk among participants:

1. Age: <45 years (0 p); 45–54 years (2 p); 55–64 years (3 p); and >64 years (4 p).
2. BMI: <25 kg/m^2^ (0 p), 25–30 kg/m^2^ (1 p); and >30 kg/m^2^ (3 p).
3. Waist circumference: men < 94 cm, women < 80 cm (0 p); men 94–102 cm, women 80–88 cm (3 p); and men > 102 cm, women > 88 cm (4 p).
4. Do you usually have at least 30 min of physical activity at work daily and/or during leisure time (including normal daily activity)? No (0 p); Yes (2 p).
5. How often do you eat vegetables, fruit, or berries? Every day (0 p), not every day (1 p).
6. Have you ever taken medication for high blood pressure on a regular basis? No (0 p); Yes (2 p).
7. Have you ever been found to have high blood glucose (e.g., in a health examination, during an illness or during pregnancy)? No (0 p); Yes (5 p).
8. Have any of the members of your immediate family or other relatives been diagnosed with diabetes (type 1 or type 2)? No (0 p); Yes: grandparent, aunt, uncle, and/or cousin (3 p); Yes: parent, brother, sister, or own child (5 p).

Risk estimates for diabetes within the next decade were categorized as low risk: <7 points (1 in 100 may develop diabetes), slightly elevated risk: 7–11 points (1 in 25 develop diabetes), moderate risk: 12–14 points (1 in 6 develop diabetes), high risk: 15–20 points (1 in 3 develop diabetes), and very high risk: ≥21 points (1 in 2 develop diabetes within the next decade) [34]. To fit data into a logistic regression analysis, we dichotomized the risk score categories by collapsing low and slightly elevated together and coded them as (0), while moderate, high, and very high risk were collapsed together and coded as (1).

## 3. Covariates of Interest

Variables in the questionnaire included physical activity, diet and self-reported health, self-reported weight, duration of residence in Norway, and sociodemographic variables. In addition, we measured weight, height, and waist circumference (WC). Body mass index (BMI) was computed from measurements taken by screening. Body mass index was calculated as weight (kg) divided by height squared (m^2^). Obesity was defined as body mass index (BMI) ≥ 30 kg/m^2^ and overweight as BMI ≥ 25 kg/m^2^ <30 kg/m^2^. A waist circumference (WC) ≥ 88 cm was considered abdominally obese. Participants were asked about the number of years they have lived in Norway, which was later categorized as ≤5 years (coded as 1), 6–10 years (coded as 2), and ≥11 years (coded as 3).

## 4. Statistical Analysis

Data was analyzed using RDS analyst version 7 and an SPSS version 19.0 statistics program. Population estimates were computed using a Giles sequential sampling estimate [35], and a multivariate analysis was performed using SPSS, while proportions of people with different sociological and demographic characteristics were also calculated. We performed a chi-square test for the analyses of categorical variables and a *t*-test for continuous variables. In order to determine the association between exposure and outcome variable, a multivariate logistic analysis was performed. The association was assessed by using a 95% confidence interval (CI) and an odds ratio (OR), with the level of significance being determined at a *P* value < 0.05.

## 5. Results

A total of 302 Somali women at the age ≥25 were interviewed from September to November 2014. The mean age of the study population was 36.13 ± 8.0 SD, whereas the mean years of residence in Norway were 9.9 ± 6.4 SD. The majority of the study population (70%) had primary or no education, with only 6% having college or university education level. Almost 45% of study participants had lived in Norway ≥11 years, while 31% and 24% had lived in Norway for ≤5 years and 6–10 years, respectively (Table 1). Regarding body mass index, the mean was 28.8 (SD 5), with 22% of participants having a normal BMI (<25); 43.2% were classified as overweight (25–29.9) and 35.2% as obese (≥30). As for waist circumferences (WC), the mean was 102 (SD 13), with 84% having a WC above 88 cm (abdominal obesity). Among the participants, 13.6% engaged daily in 30 minutes of physical activity, while 86.4% did not engage in such activity. As regards the risk of diabetes, 41% of the study population had either a moderate or high risk of developing T2D in the next 10 years. Participants with a moderate or high risk of T2D reported a significantly higher BMI (*P* = 0.001) and higher WC (*P* = 0.001) compared to those with a low/slightly elevated risk of diabetes. They were also more likely to rate their weight as heavy or very heavy (*P* = 0.001).

The risk for T2D among Somali women increases with increased age and increased years of residence in Norway. As shown in Table 2, when we treated age and years of stay in Norway as continuous variables, significant mean differences in age and years of stay in Norway were found among those who were at a low or slightly elevated risk, those who were moderately at risk, and those who were at a high or very high risk for developing T2D in the next 10 years. The mean age and years of residence in Norway are 35.7 and 9 years, respectively, among those with a low risk of diabetes. Contrastingly, the mean age was 37, and the mean years of stay in Norway were 10 years for those who were moderately at risk for diabetes. The mean age of those who were at high risk for diabetes was 38.3, and the mean years of residence in Norway were 12 years.

The age-adjusted logistic regression model (Table 3) showed that T2D risk was more common among immigrants who had a longer duration of residence in Norway. A large proportion of the excess diabetes risk was attributable to larger waist circumference, large BMI, and first-degree family history of diabetes. However, years of stay in Norway was a risk factor for T2D independently of FINDRISC factors (OR: 2.16, 95% CI: 1.01–4.62) (Table 3). The likelihood of getting T2D increased with an increase in BMI, with the obese group being nine times more likely than the normal-weight group to get the disease (OR: 9.87, CI: 3.94–24.7). The group with no rigorous physical activity was two times as likely as their counterparts practicing physical activity up to five hours a week to be at risk for T2D (OR: 2.34, CI: 1.00–5.50), an association that turned out to be significant after it was adjusted for age. The likelihood of getting T2D was significantly associated with having hypertension (OR: 7.32, CI: 1.95–27.5) and hyperglycemia (OR: 19.14; CI: 5.67–66.9). Having a family member with diabetes was significantly associated with the risk of getting T2D, and the closeness of the family member seems to influence the risk level. Respondents with an immediate diabetic family member were almost 71 times more likely than their counterparts with no diabetic family member to be at risk for T2D (OR: 70.9, CI: 27.3–183.7). Immigrants who perceived their weight as heavy were two times as likely as those who considered their weight as normal to be at risk for T2D (OR: 2.42, CI: 1.40–4.20).

We observed that consumption of fruits/vegetables did not significantly contribute to the risk of getting T2D (OR: 0.89, CI: 0.55–1.44). However, the longer duration of stay in Norway (≥11 years) remains to be significantly associated with the risk for type two diabetes even after sociodemographic and acculturation factors were adjusted (OR: 2.84, CI: 1.19–7.12) (Table 4).

## 6. Discussion

The study used a respondent-driven sampling to collect data about risk for diabetes among Somali women in Oslo area. The result shows that 41% of Somali women in the study had either a moderate/high or extremely high risk of developing T2D in the next 10 years. People at high risk for type 2 diabetes may develop prediabetes where the glucose level in the blood is higher than normal but not high enough to diagnose diabetes. For these people, specific interventions, including changes in lifestyle, have demonstrated effectiveness in reducing the incidence of diabetes [36]. The findings of this study are consistent with other studies that found that Somali immigrants have a high prevalence of diabetes [24]. In Norway and other developed nations, diabetes and obesity are markers for inequalities in health with minority populations, low-income communities and immigrants (especially as time passes since arrival) are disproportionately afflicted [37, 38]. Access to comprehensive, quality health care services is important for the achievement of health equity and for increasing the quality of a healthy life for everyone in Norway. In a fair health care system, equal needs are assumed to lead to an equal utilization of services. However, some immigrant groups are disadvantaged from achieving an equal utilization of services because of their social position or other socially determined factors, which impacts their ability to reach their full potential, thus negatively affecting their quality of life. A recent qualitative study among Somali women in Oslo shows that despite having a good knowledge about diabetes, most women reported that not only are they at risk for diabetes, but given their unhealthy lifestyle they are also incapable of avoiding it, a desperate situation that is largely attributed to the absence of a tailored preventive structure [28]. Although Norway is among countries with a fairly high social and political tolerance among minorities, preventive health services in the country rely on cultural homogeneity and universal preferences for independent learning, which may generate health inequality. A tailored intervention implies the development of health messages and facilities that are consistent with the characteristics, needs, and cultural beliefs of a given community [39]. The provision of tailored preventive services may motivate Somali women and others with similar needs toward engaging in optimal health practices and ultimately prevent them from exposure to diabetes risk.

The study found that an increased duration of residence in Norway among Somali women is significantly associated with an increased risk of diabetes. This finding is consistent with prior findings that diabetes prevalence increases among immigrants with an increased duration of residence in the host country, independent of age and body mass index (<5-year duration) [37]. In line with this, another study found an increase in the prevalence of diabetes and hypertension among immigrants to about half that of the local population within 15 years, thereby becoming equal to that of the local population after a time lag of 20 years [40]. Research has constantly found a “healthy immigrant effect”—immigrants' health is generally better than that of the host communities, although it tends to decline as their years in the host country increase. The diminished “healthy immigrant effect” with increased years of residency in the host country was suggested to be due to a transition in lifestyle behaviors [41]. A study on mortality among immigrants by cause found that Somalis experience a higher risk of death, particularly from cerebrovascular diseases, of which its main risk factors are diabetes, hypertension, and obesity [42]. A recent qualitative study that explored Somali women's utilization of preventive health services with regard to diabetes found that despite Somali women's good knowledge on diabetes, its risk factors, and preventive measures, women lack access to tailored preventive services [28], and this may explain women's risk for diabetes, which increases with an increased duration of residence in Norway. Somali women might have migrated from areas of a high level of physical activity and home grown vegetables to Norway, where they walk less, ride more, watch television more, and eat a diet higher in fat and sugars.

The largest proportions of modifiable risk for diabetes in this study were contributed by high BMI and high waist circumference, which coincides with high prevalence of sedentary lifestyle. The FINDRISC validation study indicates that increased waist circumference and BMI significantly increases type two diabetes risk [34]. A prior study found a high prevalence of central obesity among immigrants, which is independently associated with six or more years of being in the host country [22]. A prior study in Oslo suggested that the BMI change over time may be due to the limited access to a tailored physical exercise structure and, most importantly, a lack of proper health information to help them acquire adequate physical exercise [28]. The increased risk of diabetes with increased BMI and WC, which is found by this study, coincides with the fact that 86% of the participants in the study do not engage in physical activity. This finding is supported by previous research on Somali immigrants in New Zealand and Norway, which found an increased frequency of obesity, with the reason being a decrease in physical activity (PA) after immigration [22, 39]. Our finding also concurs with a prior study, which found an association between diabetes and obesity among African diaspora, which was explained by decreased levels of physical activity [5]. According to the latest WHO statistics (2008), obesity is rare in Somalia; approximately 3.1% of women and 6.4% of men were obese in 2008 [43]. Similarly, Somalia has the lowest prevalence of diabetes in Arab League countries [23]. Thus, the finding of this study is inconsistent with other studies reporting that migration per se increases the risk of developing T2D [44]. One way to tackle Somali women's risk for diabetes when they come to Norway would be to empower Somali women through knowledge about the risk factors for T2D, in addition to the provision of tailored preventive health services throughout the duration of their stay in Norway.

The result shows that consumption of fruits and vegetables did not show an association with diabetes risks. In the FINDRISC validation study by Lindström and Tuomilehto, the consumption of fruits and vegetables contributed little to the prediction of diabetes risk, but they still included it into the model to emphasize the importance of diet in T2D prevention [34]. The finding that the risk for diabetes was not associated with diet, which is found by our study, is consistent with a recent study in Sweden that found that sociodemographic factors are more important in a higher BMI among Somali women than diet [45]. Nonetheless, 50% of the participants in this study had a lower intake of fruits and vegetables. A lower intake of fruits and vegetables and high intake of meat and eggs were associated with being overweight and obese among Somali refugees in the US [46]. Similarly, uncertainty about what constituted a healthy diet, as well as a desire for education on nutrition, was reported from Somalis in the UK [47]. Therefore, an increased awareness of the association between an unhealthy diet and diabetes is vital for Somali women in Norway, as is increasing their knowledge of the available healthy diet that they may not be familiar with.

The important limitation of this study is its cross-sectional design, hence making it difficult to establish the causes. Moreover, most of the variables were self-reported, with a distinct possibility of both under- and/or overreporting. For instance, the self-reported risk for diabetes may not be a reliable source of information; however, a self-reported weight and measured weight were consistent in this study, which increase the trustworthiness of self-reported information. The high proportion of Somali women who are estimated to develop diabetes within the next decade will generate considerable diabetes-related costs owing to increased health care costs as well as productivity loss among Somali women. This probable epidemic will also reduce the quality and duration of life in this population. Thus, culturally adapted intensive prevention strategies targeting immigrant women from high risk groups may be warranted. Moreover, the findings of this study call attention to the importance of a greater understanding of the distribution of diabetes risks and processes, leading to risks among different immigrant groups in Norway. This can be done by oversampling immigrants in national diabetes studies and by inquiring about subgroup identification to help detect subgroups with an increased risk on diabetes and risk factors that this particular group may engage in, which may lead to intervention. Therefore, a qualitative research would be the next best step, which would provide us with an in-depth analysis of why Somali women are less likely to benefit from diabetes prevention services. Was it because of a lack of knowledge, a lack of access to tailored services, no information of the available services, or the respondent's beliefs? Such a study would also inform appropriate interventions, so that all individuals at risk for diabetes would benefit from national diabetes prevention services.

## Figures and Tables

**Table 1:** Differences in diabetes risk among study participants.

Indicators	Unweighted	RDS weighted	Diabetes risk		*P* value
	*N* (%)	% (CI)	Not at risk (59%)	At risk (41%)	
*Education*					
University	20 (7)	7 (4–10)	42.1	57.9	
Secondary	61 (20.7)	23 (17–29)	62.0	38.0	
Primary	138 (46.8)	45 (38–52)	58.7	41.3	*P* = 0.10
No education	76 (25.8)	25 (19–30)	70.8	29.2	

*Age*					
25–35	95 (32)	35 (27–42)	69.8	30.2	
36–45	163 (55)	53 (46–61)	60.0	40.0	
>45	37 (13)	12 (7–16)	47.2	52.8	*P* = 0.05

*Years in Norway*					
≤5	85 (30.4)	31 (25–37)	76.0	24.0	
6–10	68 (24.3)	24 (18–30)	56.0	44.0	
11+	127 (45.4)	45 (38–52)	55.0	45.0	*P* = 0.007

*Self-reported weight*					
Normal	133 (44.3)	41 (34–47)	74	26	
Heavy/very heavy	167 (55.7)	59 (53–65)	50	50	*P* = 0.001

*Body mass index*					
Normal	65 (21.6)	17.8 (0.12–0.22)	87.0	13.3	*P* = 0.001
Overweight	130 (43.2)	45.7 (0.39–0.52)	68.9	31.1	
Obese	106 (35.2)	36.5 (0.29–0.43)	34.7	65.3	

*Waist circumference*					
Normal	11 (3.7)	2 (0.03–0.4)	100	0	
Overweight	36 (12.2)	11 (0.06–15)	78.8	21.2	
Obese	247 (84)	87 (82–91)	56.2	43.8	*P* = 0.001

*Physical activity*					
Yes	41 (13.6)	15 (9–21)	73.7	26.3	
No	261 (86.4)	85 (79–90)	58.6	41.4	*P* = 0.111

*Vegetables/fruit*					
Yes	149 (49.3)	50 (42–57)	59.3	40.7	
No	153 (50.7)	50 (42–57)	62.0	38.0	*P* = 0.735

*Hypertension*					
No	283 (93.7)	94 (90–97)	63.2	36.8	
Yes	19 (6.3)	6 (3–9)	18.7	81.2	*P* = 0.001

*Hyperglycemia*					
No	263 (88.6)	88 (83–93)	67.6	32.4	
Yes	34 (11.4)	12 (0.7–17)	9.1	90.9	*P* = 0.000

*Family diabetes*					
No	163 (55.4)	53 (45–60)	90.0	10.0	
Extended family	40 (13.6)	13 (8–18)	47.4	52.6	
Close family	91 (31)	34 (27–41)	16.9	83.1	*P* = 0.000

**Table 2:** Diabetes risk by age and years of stay in Norway.

Diabetes risk score	Mean	SD
*Low or slightly elevated risk ≤11*		
Age	35	7.9
Years in Norway	9	6.7

*Moderate risk 12–14*		
Age	36.9	8.3
Years in Norway	10	6.4

*High and very high risk ≥15*		
Age	38.3	11.2
Years in Norway	12	6.3

**Table 3:** Factors associated with diabetes risk among Somali women in Oslo.

Indicators	Crude OR (CI)	Model 1 age adjusted OR (CI)
*Years in Norway*		
≤5	1.00	1.00
6–10	2.46 (1.18–5.14)	2.16 (1.01–4.62)
11+	2.58 (1.37–4.84)	2.16 (1.08–4.32)

*BMI*		
Normal	1.00	1.00
Overweight	2.93 (1.26–6.79)	2.85 (1.16–7.02)
Obese	12.23 (5.21–28.70)	9.87 (3.94–24.7)

*Rigorous physical activity over 30 minutes*		
1–5 hours per week	1.00	1.00
Do not make rigorous PHA	2.00 (0.92–4.26)	2.34 (1.00–5.50)

*Consumption of fruits/vegetables*		
Yes	1.00	
No	0.89 (0.55–1.44)	

*Hypertension*		
No	1.00	1.00
Yes	7.44 (2.07–26.7)	7.32 (1.95–27.5)

*Hyperglycemia*		
No	1.00	1.00
Yes	20.9 (6.18–70.51)	19.14 (5.67–66.9)

*Family diabetes*		
No	1.00	1.00
Extended family member	10.0 (4.35–22.9)	12,32 (4,39–34,59)
Immediate family member	44.4 (20.5–95.8)	70.9 (27.3–183.7)

*Perceived weight*		
Normal	1.00	1.00
Heavy	2.88 (1.72–4.81)	2.42 (1.40–4.20)

*Self-reported health*		
Good	1.00	
Neither good nor bad	1.66 (0.98–2.80)	
Bad	1.92 (0.81–4.56)	

**Table 4:** Association between sociodemographic variables and diabetes risks.

Indicators	Crude OR (CI)	Model 1 OR (CI)
*Age*		
25–35	1.00	
36–45	1.53 (0.86–2.69)	1.34 (0.66–2.74)
>45	2.57 (1.15–5.74)	1.52 (0.56–4.13)

*Education*		
Secondary/university	1.00	
Primary	2.00 (1.30–3.90)	1.84 (0.75–4.49)
No education	1.71 (0.91–3.19)	1.35 (0.61–2.96)

*Employment*		
Employed	1.00	1.00
Job training	0.93 (0.44–1.95)	1.14 (0.46–2.83)
Unemployed	0.96 (0.56–1.66)	1.19 (0.62–2.30)

*Years in Norway*		
≤5	1.00	1.00
6–10	2.46 (1.18–5.14)	2.74 (1.14–6.60)
11+	2.58 (1.37–4.84)	2.84 (1.19–7.12)

*Level of language*		
Fluent	1.00	1.00
Moderate	0.85 (0.45–1.61)	1.40 (0.53–3.72)
Poor	1.13 (0.58–2.21)	1.20 (0.52–2.77)

*Following local media*		
Yes, everyday	1.00	1.00
Often	1.49 (0.75–2.93)	1.89 (0.79–4.49)
Rarely or never	1.35 (0.54–3.36)	1.12 (0.41–3.09)

*Ethnicity of friends*		
Somalis, other immigrants, and Norwegians	1.00	1.00
Somalis & other immigrants	0.50 (0.27–0.90)	0.59 (0.28–1.24)
Only Somalis	1.26 (0.69–2.31)	1.20 (0.54–2.68)
